# The Active Metabolite of Warfarin (*3'-Hydroxywarfarin*) and Correlation with INR, Warfarin and Drug Weekly Dosage in Patients under Oral Anticoagulant Therapy: A Pharmacogenetics Study

**DOI:** 10.1371/journal.pone.0162084

**Published:** 2016-09-08

**Authors:** Donato Gemmati, Francesco Burini, Anna Talarico, Matteo Fabbri, Cesare Bertocco, Marco Vigliano, Stefano Moratelli, Antonio Cuneo, Maria Luisa Serino, Francesco Maria Avato, Veronica Tisato, Rosa Maria Gaudio

**Affiliations:** 1 Centre Haemostasis & Thrombosis, Section of Medical Biochemistry, Molecular Biology and Genetics, Department of Biomedical and Specialty Surgical Sciences, University of Ferrara, Ferrara, Italy; 2 Section of Medicine and Public Health, Department of Medical Sciences, University of Ferrara, Ferrara, Italy; 3 Department of Morphology, Surgery and Experimental Medicine and LTTA Centre, University of Ferrara, Ferrara, Italy; Ehime University Graduate School of Medicine, JAPAN

## Abstract

**Objectives:**

Warfarin oral anticoagulant therapy (OAT) requires regular and frequent drug adjustment monitored by INR. Interindividual variability, drug and diet interferences, and genetics (*VKORC1* and *CYP2C9*) make the maintenance/reaching of stable INR a not so easy task. HPLC assessment of warfarin/enantiomers was suggested as a valid monitoring-tool along with INR, but definite results are still lacking. We evaluated possible correlations between INR, warfarin/3’-hydroxywarfarin, and drug weekly dosage aimed at searching novel alternatives to OAT monitoring. *VKORC1/CYP2C9* pharmacogenetics investigation was performed to account for the known influence on warfarin homeostasis.

**Methods:**

133 OAT patients were recruited and assessed for warfarin/3’-hydroxywarfarin serum levels (HPLC), INR, and *VKORC1* and *CYP2C9* genotypes. A subgroup of 52 patients were monitored in detail (5 consecutive controls; c0-c4) till the target INR was reached. Correlation analyses were performed in both groups

**Results:**

In the whole OAT group both warfarin and 3’-hydroxywarfarin correlate with INR at comparable degree (*r*^2^ = 0.0388 and 0.0362 respectively). Conversely, warfarin weekly dosage better correlates with warfarin than with 3’-hydroxywarfarin (*r*^2^ = 0.0975 and *r*^2^ = 0.0381 respectively), but considering together warfarin plus 3’-hydroxywarfarin the correlation strongly increased (*r*^2^ = 0.1114; *p*<0.0001). Interestingly, 3’-hydroxywarfarin reached a strong correlation at c4 respect to warfarin (*r*^2^ = 0.2157 and *r*^2^ = 0.0549; *p* = 0.0005 and *p* = 0.0944 respectively) seeming less affected by drug adjustments in the subgroup of 52 patients who started OAT. The multivariate analyses aimed at estimating the true contribution of 3’-hydroxywarfarin on INR value ascribed it the unique significant value (p = 0.0021) in spite of warfarin who lost association. The pharmacogenetics studies confirmed that patients carrying the *VKORC1* variant-allele required lower warfarin maintenance dosage and that the combination of *VKORC1* and *CYP2C9* yielded a warfarin responsive index (WRI) inversely related to the number variant alleles

**Conclusion:**

Our results overall suggest that 3’-hydroxywarfarin monitoring could be of great advantage in INR monitoring respect to classical warfarin assessment showing significant contribution also in multivariate analysis. Therefore, additional active metabolites should be recognized and investigated as novel useful indicators.

## Introduction

Warfarin is the most commonly prescribed oral anticoagulant in the continuous prophylaxis and treatment of a number of severe thromboembolic disorders and complications [1]. The anticoagulant effect is mediated by interfering with the synthesis of vitamin K-dependent clotting factors in the liver *via* inhibition of the enzyme *VKORC1* (vitamin K epoxide reductase complex subunit 1) [2, 3]. A wide and strong interindividual variability hampers the actions required to optimize therapy in dose, as well as in requested time to stabilize patients in term of maintaining optimal and efficacious anticoagulation. Warfarin treatment needs to be frequently monitored by a laboratory test named prothrombin time (PT) conventionally expressed as the International Normalized Ratio (INR).

The introduction of INR, obtained by ISI calibration (International Sensitivity Index), has been proposed to improve INR accuracy and inter-laboratory precision accounting for several variables such as different thromboplastin used, instrumentations and reference controls. Despite of the fact that a lot of work and efforts have been applied to get target INR easy to maintain and comparable among different laboratories, significant dose adjustments are often required during therapy and the rate of warfarin-related bleeding and other events has not diminished, particularly in complicated patients [4, 5]. The main causes affecting warfarin efficacy are related to genetic factors including *VKORC1* and cytochrome P450 (*CYP*) polymorphisms [6–12], the concomitant drugs/treatments, the variations in diet and various disease states, by changing its pharmacokinetics [13–15]. Finally, patients’ noncompliance has a high impact also on dose adjustment, making the treatment strategy of clinicians highly risky. In general, low correlation has been described between warfarin circulating levels and INR, making difficult any pharmacological or clinical inference [16].

Warfarin is a racemic mixture of *R*- and *S*-enantiomers, characterized by the *S*-isoform with higher anti-vitamin-K activity than the *R*-warfarin [17]. On the other hand, *S*-warfarin has a shorter half-life because its clearance is greater than that of *R*-warfarin. In addition, the *R*-enantiomer is mainly reduced to an active 3’-hydroxywarfarin, and the S-enantiomer mainly to 6’- and 7’-hydroxywarfarin, ascribing in general to the reduced metabolites lower activity than warfarin and considering the 6’- and 7’-hydroxymetabolites inactive [18]. Several analytical methods have been developed to assess the main warfarin metabolites with clinical weight. The effective measurement of serum warfarin concentration might overcome the above described problems, for this reason, a series of completely different approaches from the INR-based monitoring have been proposed: gas chromatography–mass spectrometry (GS–MS), high performance liquid chromatography–mass spectrometry (HPLC–MS) as well as HPLC and a chiral HPLC-tandem mass spectrometry (HPLC-MS/MS) [18–22]. By means of these sensitive and specific analytical methods, it is possible to quantify warfarin and its clinically significant metabolites to improve the understanding of warfarin pharmacology/pharmacodynamics and to find out potential novel interferences, with the aim of a better monitoring of the anticoagulation range in patients. However, large part of the studies has been directed towards the assessment of warfarin and the inactive 7’-hydroxywarfarin in INR comparison analyses, while no data are available about the potential role and assessment of an active metabolite, particularly in kinetic analyses of patients starting for the first time OAT therapy until reaching stable INR. For these reasons, to investigate and reveal the relationships and correlations among INR, anticoagulant molecule concentrations and the weekly warfarin dosage, we assessed by HPLC warfarin and 3’-hydroxywarfarin serum levels in two different set of patients: a first group of patients on warfarin for long time and a second group which has only just started warfarin therapy and in which we monitored and longitudinally analyzed all the above described variables up to the INR reached the target range. Finally, pharmacogenetics and correlation studies have been performed.

## Materials and Methods

### Patient recruitment and blood samples

A total of 133 unrelated patients who received oral warfarin anticoagulant therapy (OAT) at the Haemostasis & Thrombosis Centre (HTC) of the Hospital-University of Ferrara, Italy, were assessed for warfarin and 3’-hydroxywarfarin metabolite, PT-INR, and DNA analyses. All patients were outpatients attending HTC for OAT monitoring due to venous thrombotic accidents and consisted of 81 patients receiving warfarin for long time (established-group) and the remaining 52 were at their first control at HTC (starting-group). The established-group was defined as patients having stable INR at least for three different controls ranging at least 9–12 weeks. The starting-group attended ambulatory at HTC within three-days from the first drug assumption consisting of a standard dose of 5 mg/day. Patients were selected from a whole group of 600 outpatients from those (n = 3500) attending the HTC ambulatory as previously reported [23]. Exclusion criteria were allergic reactions to warfarin, bleeding tendency, abnormal renal/liver function, use of aspirin, barbitals and quinidine, cardiovascular diseases. Finally, limited to the starting-group, patients with INR-values above 1.49 at the first control (c0) were not included. The male/female ratio was 50% in both groups and the mean age was 64.5±14.5 (range 55–75). The study followed the local Institutional Review Board guidelines for investigation on human biological samples. In this case, the laboratory processed completely anonymous blood samples labeled by a barcode [24]. The automated coagulometer (ACL TOP 700 CTS and 700 LAS, Werfen Company) records and matches INR results coupled with the unique barcode. Accordingly, after therapy prescription, dedicated software for OAT managements (P.A.R.M.A., *Automated Program for Oral Anticoagulant Treatment*; Werfen Group) gives the INR values and the date of the next control, and patients are identified only through a numerical sequence. For the described procedure none of the authors had access to the identity of patients. So, both laboratory and clinical staff worked in blind and anonymization occurred before the authors had access to blood samples. No IRB approval was needed, because of the study did not modify the clinical and/or therapeutic management of patients. It was merely based on the extraction and computing of routine data of the coagulation analysis (INR) and of the warfarin(s) assessment by HPLC. Accordingly, the permission to perform additional tests (HPLC) was asked to patients and those who agreed signed informed consent to enter the study. In this case the clinical staff responsible for blood sampling was provided by an additional sticky label reporting the associated personal bar-code. Blood samples, for INR evaluation (vacutainer® sodium-citrate tube, BD), warfarin HPLC assessment (Greiner bio-one, vacuette® Z Serum Sep Clot Activator) and DNA analyses (vacutainer® K2EDTA tube, BD) were drawn contextually to the same day of the patient’s control. Plasma or serum separation, as well DNA extraction and storing were as previously reported [25–27].

### Warfarin and 3'-OH-warfarin assessment by HPLC

The analysis was performed as previously described [28, 29] with few modifications, by using a HPLC system with UV detector and ternary isocratic pump (Merck Hitachi) with C18 column (Omnisphere C18 25 cm x 4.6 mm, 4.6 μm). Warfarin sodium and its major active metabolite 3'-hydroxywarfarin were directly provided by Bristol-Myers Squibb Company whilst rodenticide Coumatetralyl was used as internal standard (IS; Riedel-de Haën, Sigma Aldrich, Laborchemikalien GMBH). The mobile phase for the system H/Z consisted of 146 μL of triethanolamine (TEA) to which were added 750 μL of concentrated phosphoric acid, 530 mL of H_2_O for HPLC; after reaching the pH 3.3 by mean of KOH 10M, 620 mL of CH_3_CN were added. The running conditions for the system H/Z were at a wavelength of 280 nm and a flow rate 1mL/min.

Blood was collected into vacuum tubes (Greiner bio-one) type Z-serum-sep-clot-activator. Blood specimens were centrifuged at 200g for 10 minutes at room temperature and stored at -80°C until testing. The system H/Z must be conditioned with a solution of CH_3_CN:H_2_O (1:1). This is followed by the analytical conditions above reported. 30 μL of IS solution were placed in a vial and air-dried, then 1mL of test serum was added and mixed by vortexing; then 350 μL of 1M H_2_SO_4_ and 4 ml of (C_2_H_5_)_2_O were added and mixed again. After a fast spinning step the supernatant organic phase was transferred to a second vial and air-dried. The precipitate was reconstituted with 70 μL of mobile phase and 20 μL of it were injected in the H/Z system.

For calibration curves, warfarin and 3'-hydroxywarfarin purified molecules were treated in the same manner. The equations describing the two curves are the following: warfarin: Y = 0.5216X–0.0091 (Y = Area warfarin/Area IS); *r*^2^ = 0.994; 3’-OH-warfarin: Y = 0.8243X–0.0762 (Y = Area 3’-OH-warfarin/Area IS); *r*^2^ = 0.994

**Fig 1:**
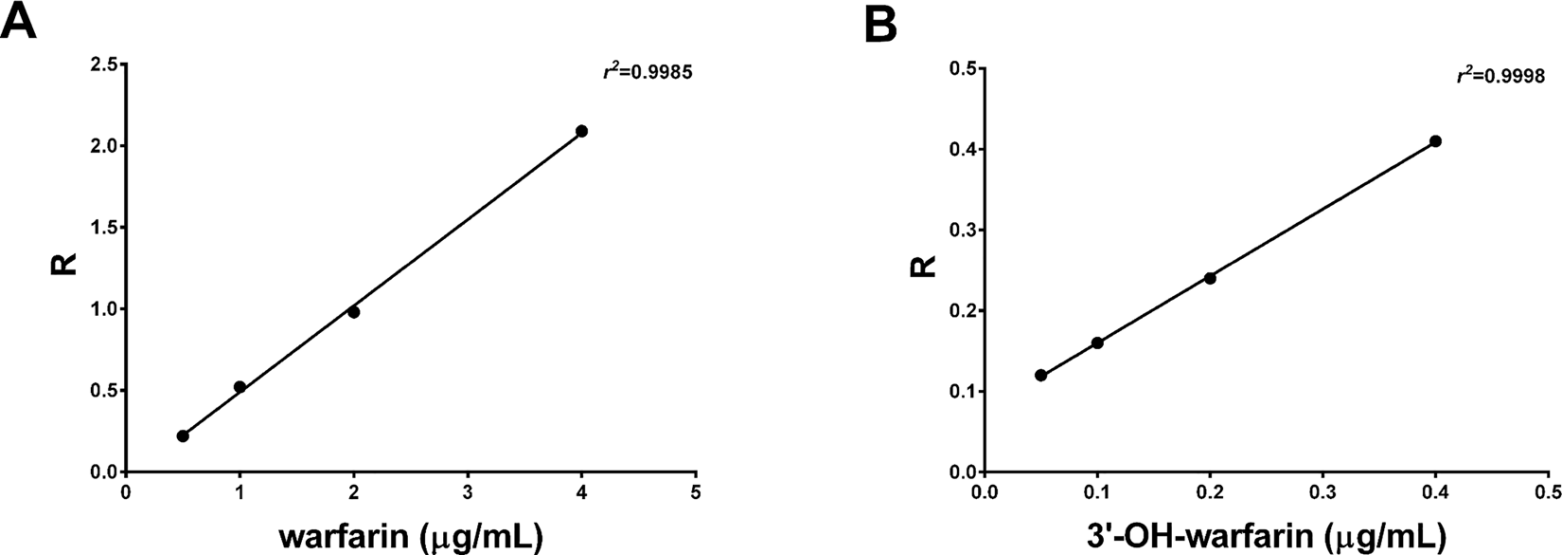
HPLC calibration curves. Warfarin (1A) and 3’-hydroxywarfarin (1B) calibration curves; R expresses the ratio between the area under the analyte peak (warfarin or 3’-hydroxywarfarin respectively) and the area of the internal standard.

Fig 1 shows the calibration curves respectively for warfarin and 3’-hydroxywarfarin (Fig 1A and 1B respectively). Linearity was excellent for both curves (*r*^2^ = 0.9985 for warfarin and *r*^2^ = 0.9998 for 3’-hydroxywarfarin).

### DNA analysis of candidate SNPs

Genomic DNA was isolated using QIAamp DNA Mini Kit (Qiagen). Multiplex PCR amplification was as previously described [19] and Table 1 shows the complete primers set utilized. For the SBE-genotyping (Single Base Extension), we designed four different primers. PCR amplification was performed on a PCR thermal cycler (model Verity 96-Well Fast, Applied Biosystems) using the following conditions: 5 min of denaturation at 94°C, followed by 35 cycles of 30s at 94°C, 30s at 55°C, and 45s at 72°C, with a final extension at 72°C for 5min. The PCR products were purified by incubation at 37°C for 15 min with 5U of shrimp alkaline phosphatase (SAP) and 2U of Exonuclease I (ExoI) and finally inactivated by incubation at 85°C for 15 min. The SNaPshot Primer Extension Reaction Mix (5μL) was mixed with 3μL of purified PCR DNA template, in a final reaction volume of 10μl (according to ABI Prism SNaPshot Multiplex kit; Applied Biosystems). About 0.2μM each of the three primers was included in the same tube. A standard reaction was also run with SNaPshot multiplex control primer mix and SNaPshot Multiplex control template DNA. The SBE primers were extended in a PCR thermal cycler (model Verity 96-Well Fast, Applied Biosystems) as follows: 10s at 96°C, 5s at 50°C and 30s at 60°C for 25 cycles. After primer extension, each reaction was treated with 1U of FastSAP (Fermentas) for 10 min at 37°C, followed by a 5 min incubation at 75°C for SAP inactivation. The amplified fragments were separated using an ABI Prism 310 DNA Genetic Analyzer (Applied Biosystems) following the manufacturer’s recommendations, a POP-4 polymer in conjunction with the GS POP-4 (1 mL) E5 module. Following the run, samples were analyzed using GeneMapper ID-X 1.1 software (Applied Biosystems).

**Table 1:** PCR multiplex amplification of DNA for primer extension.

CYP2C9*2 (C430T)	
CYP2C9*2-Fw	5’-GTATTTTGGCCTGAAACCCATA-3’ (22 mer)
CYP2C9*2-R	5’-ACCCTTGGTTTTTCTCAACTC-3’ (21 mer)
CYP2C9*3 (A1075C)	
CYP2C9*3-Fw	5’-TGCACGAGGTCCAGAGATGC-3’ (20 mer)
CYP2C9*3-R	5’-GATACTATGAATTTGGGGACTTC-3’ (23 mer)
***VKORC1* (-C1173T)**	
*VKORC1*-Fw	5’-AAAAGCAGGGGCTACG-3’ (16 mer)
*VKORC1*-R	5’-CCGAGAAAGGTGATTTCCA-3’ (19 mer)
**SNaPshot primer extension**	
CYP2C9*2 (C430T)	5’-(T)20GGAAGAGGAGCATTGAGGAC-3’ (42 mer)
CYP2C9*3 (A1075C)	5’-(T)20ACTGCTGGTGGGGAGAAGGTCAA-3’ (48 mer)
***VKORC* (-C1173T)**	5’-(T)11TGCCCGTGTCCAGGAGATCATCGAC-3’ (36 mer)

### Statistical analyses

A simple linear regression model was performed to assess relationships among warfarin, 3’-hydroxywarfarin concentrations, weekly/day warfarin dosage, and PT-INR. Mean, median, SD and range were calculated for warfarin/3'-hydroxywarfarin serum concentrations, INR, and warfarin dosages. Casual genotypic distribution (*VKCORC1* and *CYP2C9*) in the groups of patients was verified by using Chi-square test (Hardy-Weinberg equilibrium test). To assess differences in INR, warfarin, 3'-hydroxywarfarin serum concentrations or in drug weekly dosage, the cohort of patients has been stratified by *VKORC1* genotype and *t*-Student test was performed. Kruskall-Wallis test was used to evaluate significant differences of daily warfarin dosage requirement in the different categories according to warfarin responsive index (WRI) accounting also for *CYP2C9* genotypes [30]. Finally, correlation coefficients for warfarin, 3’-hydroxywarfarin concentrations, weekly/daily warfarin dosage, and PT-INR were tested with Spearman-correlation test. Multivariate analyses by multiple regression models were applied to estimate the true contributors of 3’-hydroxywarfarin to INR values. To consider a patient “out-of-range” in INR value after a starting period to reach the ideal INR target (2.0–3.0), we applied a minimum threshold value (INR<1.99) and a maximum value lower or equal to 3.35 (95th percentile of our whole cohort of stabilized patients). *p*-values less or equal to 0.05 were considered statistically significant. Statistical analysis was done by SPSS (version 21, Chicago, IL) and GraphPad Prism (version 5, La Jolla, CA) software.

## Results

### Correlations between warfarin(s), INR, and warfarin weekly dosage in patients on established warfarin therapy

**Fig 2:**
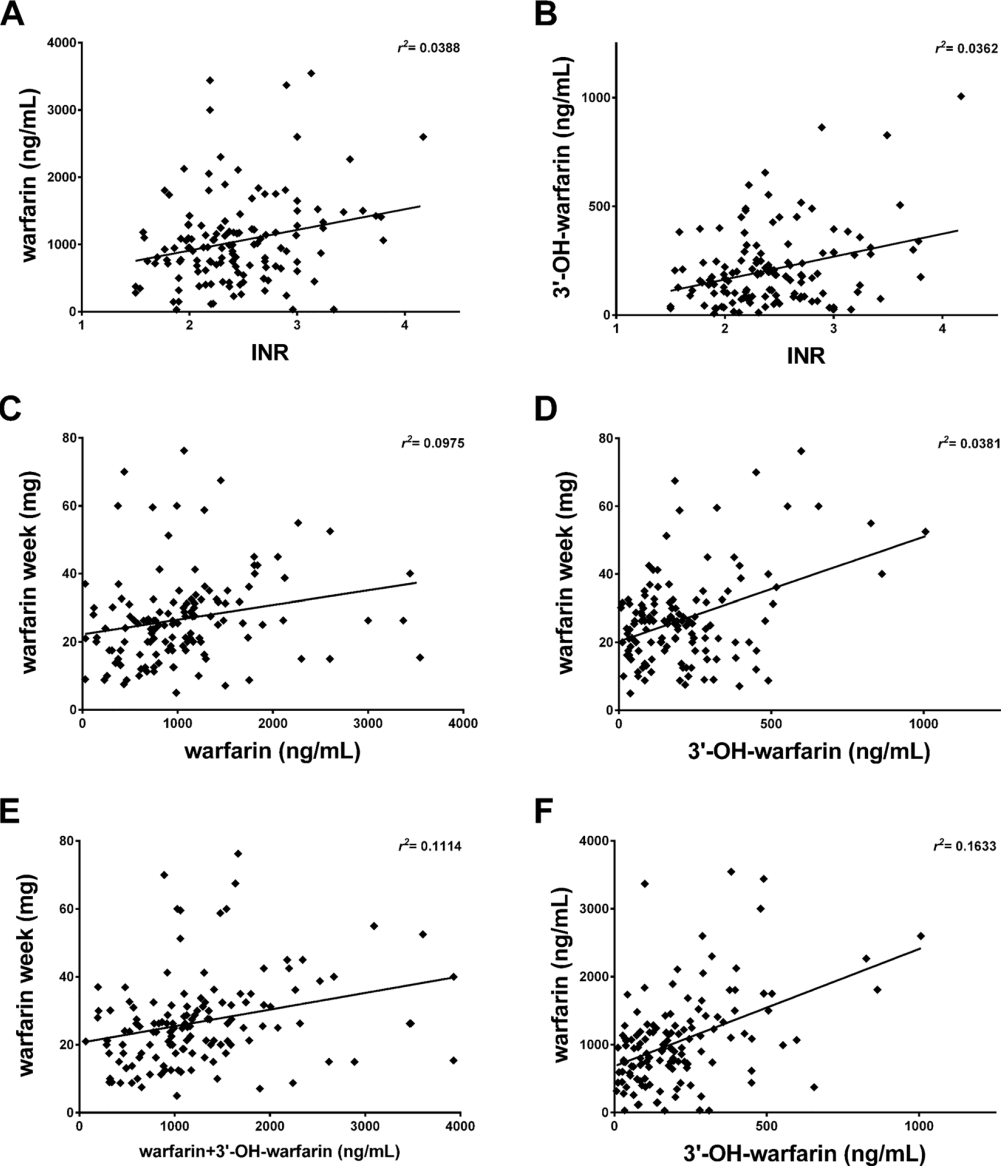
Correlation analyses between: INR, serum warfarin (ng/mL) and 3’-hydroxywarfarin (ng/mL), and the amount of drug (warfarin week) taken by the whole cohort of patients on oral anticoagulant therapy.

Fig 2A shows the results of the correlation between serum warfarin concentration and INR among the 133 patients with established anticoagulant treatment. The correlation reached appreciable significant values (*r*^*2*^ = 0.0388; *p* = 0.023). At the same extent, the serum 3’-hydroxywarfarin correlated with INR (Fig 2B; *r*^*2*^ = 0.0362; *p* = 0.028) and the strength increased when warfarin and 3’-hydroxywarfarin were computed together (*r*^*2*^ = 0.0455; *p* = 0.0137, graph not shown). Considering warfarin weekly dosage, it better correlates with warfarin (Fig 2C) than with 3’-hydroxywarfarin (Fig 2D) (*r*^*2*^ = 0.0975 and *r*^*2*^ = 0.0381 respectively), but considering together warfarin plus 3’-hydroxywarfarin the correlation strongly increased (Fig 2E; *r*^*2*^ = 0.1114; *p*<0.0001). In addition, the strongest correlation found was the one that analyzes warfarin versus 3’-hydroxywarfarin concentrations (Fig 2F; *r*^*2*^ = 0.1633; *p*<0.0001). Finally, the warfarin weekly dosage did not reveal any significant correlation with the INR values, (*r*^*2*^ = 0.0041; *p* = 0.463, graph not shown).

### Monitoring of INR, warfarin and 3’-hydroxywarfarin levels in patients who started warfarin therapy (c0-c4)

**Fig 3:**
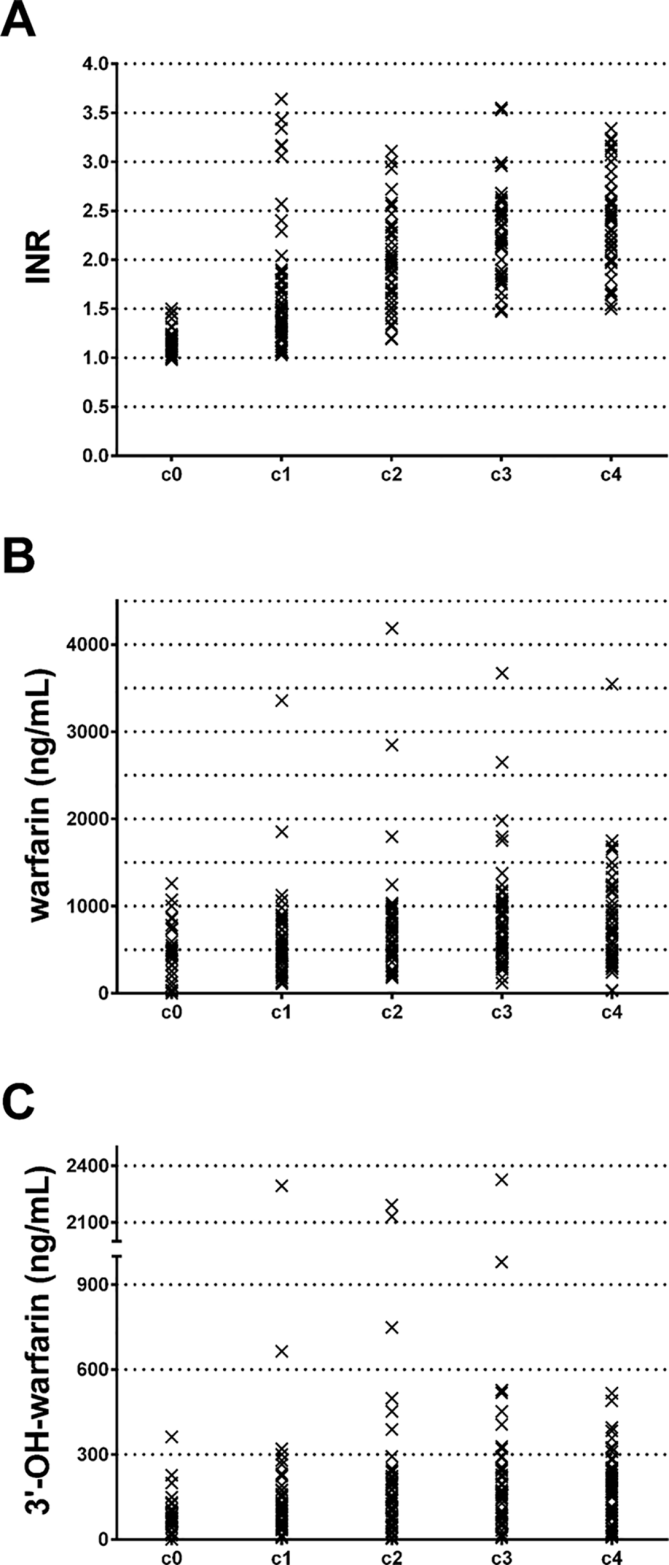
Fluctuations of INR (A), warfarin (B) and 3’-hydroxywarfarin (C) serum concentrations among the subgroup of patients which starts oral anticoagulant therapy, during the time frame of 17–57 days (c0-c4).

Fig 3A, 3B and 3C, show the dynamics of INR values, serum warfarin and 3’-hyroxywarfarin concentrations during the period ranging from the first control (c0) to an hypothetical frame of time long enough to reach a stable INR target (target: 2.0–3.0). This period of time globally encompassed a mean of 29.38±8.2 days (range, 17–57) after which a low percentage (11.54%, at c4) of patients had INR out of range. It is of note that c1 shows the widest INR range, including patients either with unmodified INR and others with INR>3.0 (Fig 3A). Conversely, the fluctuations in warfarin concentrations, with rare exceptions, were slighter if compared with those of INR at the same frame of time considered (Fig 3B). As the 3’-hyroxywarfarin dynamic is concerned, it was characterized by appreciable changes starting from c2 and stabilizing at c4 (Fig 3C).

A detailed schedule of the intermediate-times, referred to the consecutive controls, is shown in Table 2 and the specific INR values at c0, c1, c2, c3 and c4 (mean ± SD, median and range) are compared.

**Table 2:** Time-frame findings in patients starting OAT (n = 52).

day interval	c0-c1	c1-c2	c2-c3	c3-c4	Total period c0-c4
***mean±SD***	4.52±1.04	6.19±2.86	7.76±4.14	12.63±6.78	29.38±8.2
***median***	4.0	6.0	7.0	11.0	29
***range***	3–7	2–17	2–20	7–31	17–57
**INR *mean±SD***	1.16±0.15	1.71±0.67	2.07±0.81	2.28±0.50	2.44±0.46
***median***	1.13	1.49	1.92	2.24	2.37
***range***	0.98–1.5	1.03–3.64	1.19–5.73	1.47–4.01	1.50–3.34
***out-of-range n, (%)***	52 (100)	48 (92.31)	33 (63.46)	17 (32.69)	6 (11.54)

### Correlation between INR and warfarin(s) concentrations in patients who started warfarin therapy

On the basis of the above demonstrated correlation between INR and warfarin in stabilized patients (Fig 2A to 2F), contrasted by the absolute lack of correlation between the effective amount of drug taken by the patients and INR (*r*^*2*^ = 0.0041; *p* = 0.463), we aimed at investigating in detail how patients who started therapy reached and maintained such findings. By utilizing the same approach, we performed analyses at any scheduled control that patients had up to the target INR was presumably reached (c0-c4).

Table 3 shows the coefficients of correlations and the associated *p*-values of INR/warfarin and INR/3’-hydroxywarfarin comparisons at every scheduled controls. At the beginning of drug treatment, correlation was found at c0 only in warfarin (*p* = 0.0164) being borderline as regards 3’-hydroxywarfarin (*p* = 0.098), being this latter a metabolite of the former. Interestingly, after an initial improvement of correlation observed for both at c1, only 3’-hydroxywarfarin shows at c4 a clear strong correlation (*r*^*2*^ = 0.2157). Conversely, warfarin never reached correlation when compared with the respective INR value during the whole time-frame considered with exclusion of a borderline value at c4 (p = 0.0944). However, when computed in the whole group of stabilized patients it showed an appreciable *p*-value (*p* = 0.023). Conversely, 3’-hydroxywarfarin reaches strong correlation at c4 (*p* = 0.0005) also confirmed in the whole group of stabilized patients (*p* = 0.028). This contrasting data are probably due to the frequent drug adjustments (in terms of reduction/augmentation) necessarily affecting the dose-response correlation. It is of note the presence of warfarin and of 3’-hydroxywarfarin in serum of patients at c0, this because they often presented at our Centre after an initial standard dose administrated during the first 24–48h at the emergency examination.

**Table 3:** Correlation analysis between INR and warfarin / 3’-hydroxywarfarin in the two cohorts of patients investigated.

	*Dynamics in first-enrolled cases (n = 52)*					*Stabilized cases (n = 133)*
	c0	c1	c2	c3	c4	Total
INR (*median*)	1.13	1.49	1.92	2.24	2.42	2.37
INR /warfarin						
*r*^*2*^*-value*	0.1098	0.1449	0.0227	0.0162	0.0549	0.0388
*p-value*	**0.0164**	**0.0054**	0.2861	0.3681	0.0944	**0.023**
INR/3’OH-warfarin						
*r*^*2*^*-value*	0.0535	0.0725	0.0027	0.0087	0.2157	0.0362
*p-value*	0.0989	0.0535	0.7167	0.5102	**0.0005**	**0.0284**

Finally, to estimate the true contribution of different variables on INR levels, univariate and multivariate analyses have been performed accounting for the following variables: warfarin, 3’-OH-warfarin, warfarin weekly dosage, *CYP2C9* genotype and *VKORC1* genotype as shown in Table 4. Interestingly, at univariate analysis both warfarin and 3’-OH-warfarin reached statistical significance while at multivariate analysis only 3’-OH-warfarin maintains high statistical significance (*p* = 0.0021).

**Table 4:** Univariate and multivariate analyses to estimate the contribution of different variables on INR.

Variables	*p-value* (univariate)	*p-value* (multivariate)
Warfarin (ng/mL)	**0.0057**	0.1421
3’-OH-warfarin (ng/mL)	**0.0002**	**0.0021**
Warfarin weekly dosage	0.9431	0.0764
*CYP2C9* genotype	0.2261	0.3175
*VKORC1* genotype	0.9512	0.8663

### Genotype correlation studies

We additionally investigated possible influences on the warfarin dynamics due to the different combination of genotypes of the four SNPs in the two different genes investigated [i.e. CYP2C9*2 (416 C>T); CYP2C9*3 (1061 A>C); *VKORC1* (-1173 C>T), and *VKORC1* (-1639 G>A)].

**Fig 4:**
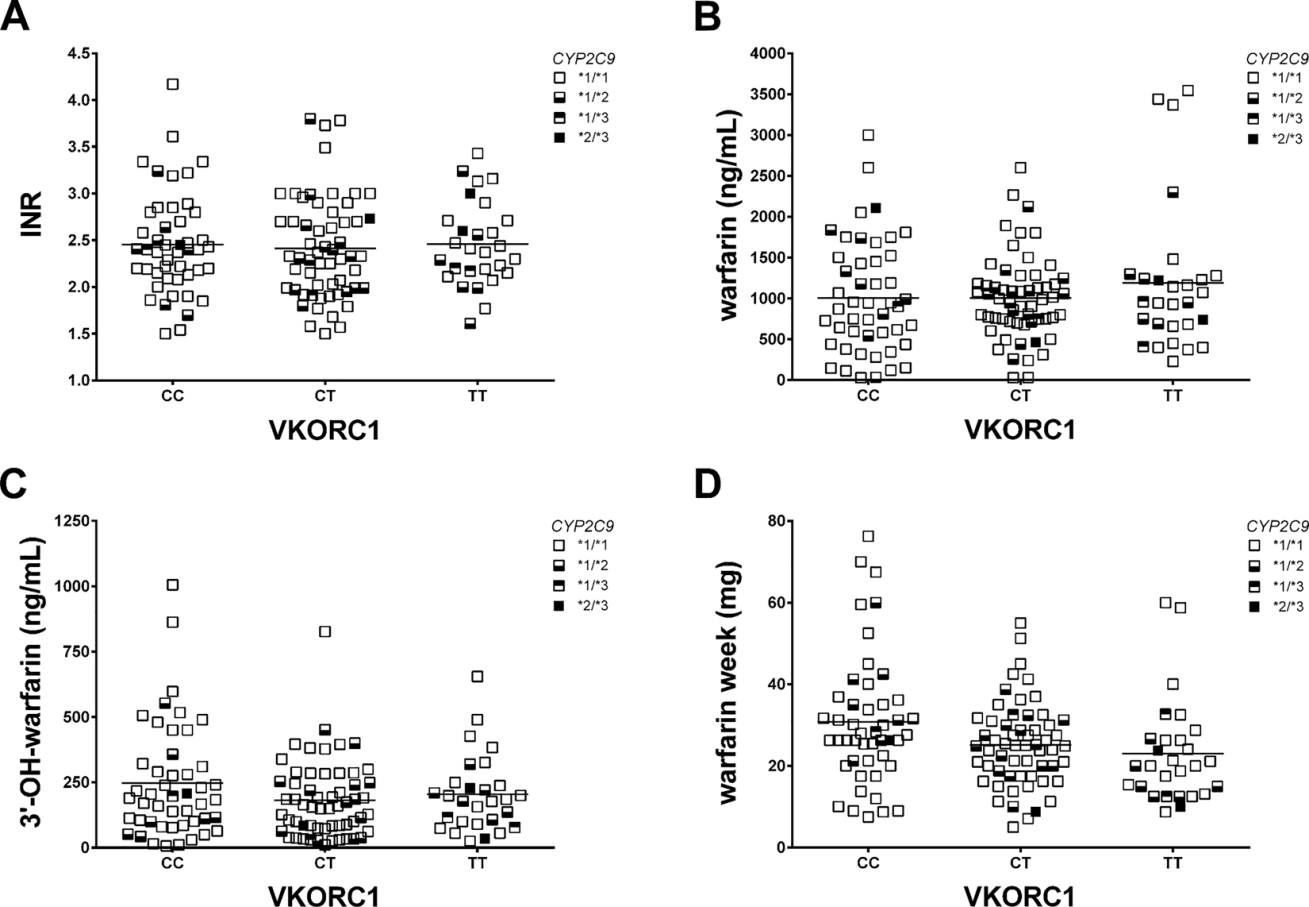
Genotype distributions in the whole cohort of patients. Different distributions of *CYP2C9* haplotypes in the whole cohort of patients stratified by the three *VKORC1* genotypes according to INR values (A), warfarin and 3’-hydroxywarfarin serum concentration (B, and C respectively), and warfarin week (D).

For this purpose, in the whole group of stabilized patients, the INR values, the warfarin and 3’- hydroxywarfarin concentrations, and the weekly amount of warfarin drug were stratified for the three different *VKORC1* classes of genotypes (i.e. -1173 CC, CT and TT). To exclude any INR interference on the results, we assured that the three *VKORC1* classes of genotypes had comparable INR distributions. Neither significant difference nor correlation was found among the three classes of *VKORC1* genotypes and INR (Fig 4A), as well no differences were obtained considering 3’-hydroxywarfarin distribution, though wider ranges were observed among the CC-subgroup (Fig 4C). Similarly, mean warfarin concentrations did not yield significant differences among the three *VKORC1* genotypes (Fig 4B), also when *VKORC1–*1173 T-carriers (CT and TT) were compared with the not-carriers cases (-1173 CC-homozygotes). Interestingly, stratifying the amount of taken warfarin (warfarin mg/week) by *VKORC1* genotypes, the amount of warfarin/week significantly decreased as the number of *VKORC1*–1173 T-alleles increased in the genotype of patients (Fig 4D; *p*-trend = 0.026; *r*^*2*^ = 0.546). This means that patients with the CC *VKORC1* genotype need a significantly higher mean daily warfarin maintenance dosage than those with the TT genotype (4.39±2.29 mg and 3.29±1.82 mg respectively; *p* = 0.033), whilst the CT genotype was characterized by an intermediate mean daily warfarin dose. As known, the *VKORC1*–1639 G>A, and the -1173 C>T polymorphisms are in complete *linkage disequilibrium* (-1173T and -1639G) and for that reason we showed just a single stratification analysis.

**Fig 5:**
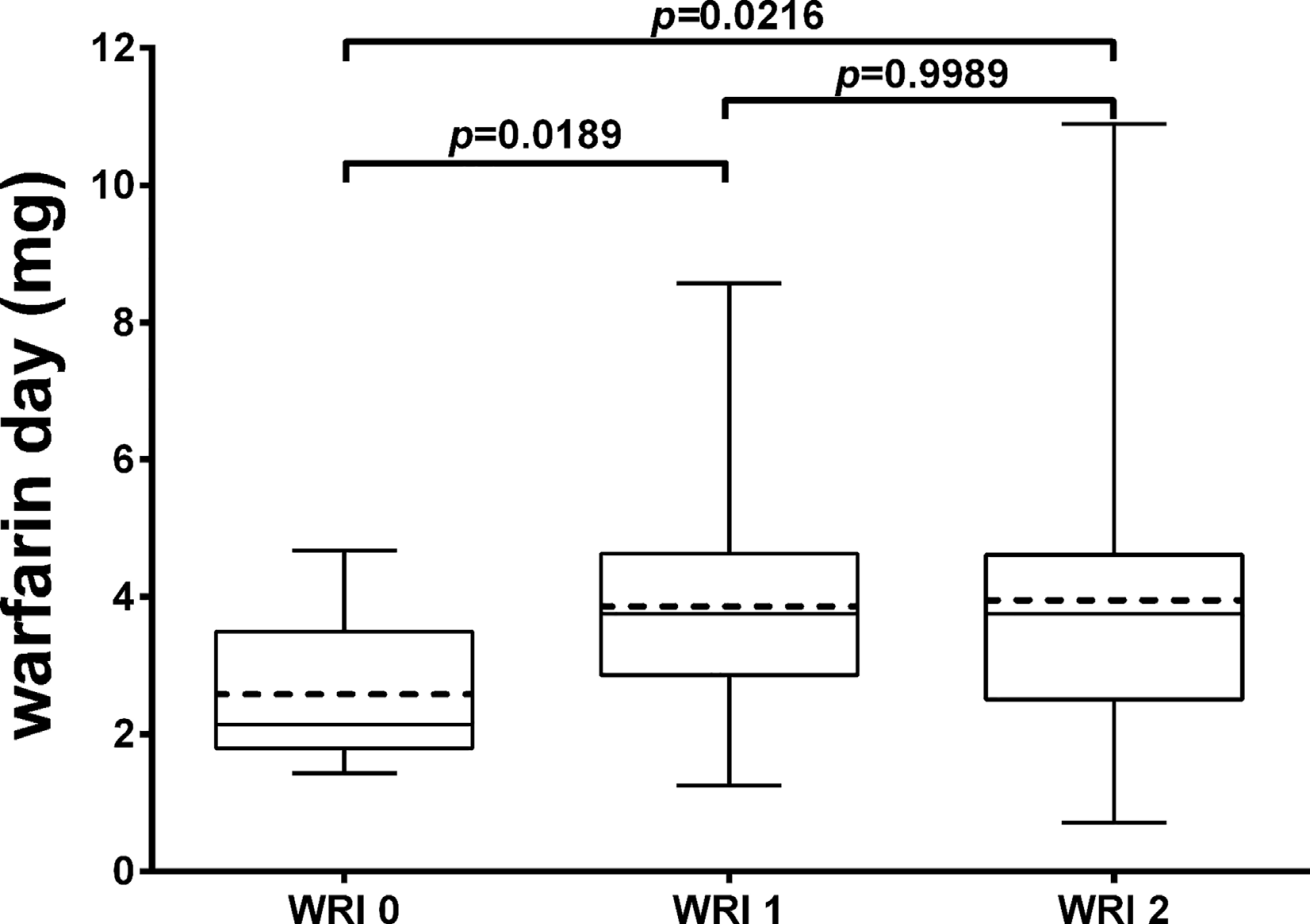
Warfarin daily dose for different WRI. Mean and median warfarin dose increased as WRI increased. WR 0, WRI 1 and WRI 2 classes are as specified in text. Continuous line indicates the median; dashed line indicates the mean, vertical bars indicate the 1^st^ and 99^th^ percentile of warfarin day (mg).

Although the CYP2C9*1/*2/*3 polymorphisms were equally distributed among the three different *VKORC1* classes of genotypes (data mainly obtained by the *1/*1 haplotype status), we performed a sub-analysis aimed at investigating the effects of the two genes on the dose of warfarin required. By using the warfarin responsive index (WRI) as previously described [30] we assigned a WRI score of 1 to *VKORC1* CC or CT, and a score of 0 to TT. For the CYP2C9*1/*2/*3 polymorphisms we assigned a WRI score of 1 to CYP2C9*1/*1 and a score of 0 to the remaining sub-classes. Accordingly, Fig 5 shows that the mean of warfarin daily dose varies among the three different WRI categories (WRI 0 vs WRI 1 + WRI 2; *p* = 0.0157), ascribing to the group 2 a significant higher dosage (WRI 2 *vs* WRI 0, *p* = 0.0216; WRI 1 *vs* WRI 0, *p* = 0.0189). As a result, patients belonging to the three different WRI categories varied significantly in drug requirement (2.58±1.06, 3.87 ±1.743, and 3.95±2.0 mg/day for the WRI scores of 0, 1, and 2 respectively; *p*-trend = 0.05).

## Discussion

Although, the introduction in the clinical practice of the novel anticoagulants (NOA), differing from warfarin mainly for the lack of laboratory monitoring or dose adjustments, several issues still exist about these new substances (e.g. limitation in therapeutic eligibility, absence of specific antidotes). In addition, patients often might go back, transitionally or definitively, to warfarin treatments [31–33]. For these reasons, it is plausible that traditional OAT will retain its wide treatment application in the antithrombotic prophylaxis and therefore continuous investigations in the laboratory management are warranted.

In this work we mainly investigated the mutual relationships between the INR value and the concentration of warfarin, 3-hydroxywarfarin or the weekly dosage administered to patients on OAT. In addition, pharmacogenetics of the main SNPs involved in the associated drug homeostasis have been taken into account.

Large part of the studies present in literature has been directed towards the assessment of warfarin and the inactive 7’-hydroxywarfarin in INR comparison analyses missing the possibility to investigate unrevealed roles of active metabolites. In particular, active metabolites might better account for the different basal activity and half-life of *R*- and *S*-enantiomers. Finally, no data are available in kinetic studies for active metabolites in patients starting for the first time OAT until reaching stable INR.

First of all, in our cohort of stabilized patients, the warfarin weekly dosage did not reveal any correlation with the INR values, raising the old issue about the right method to monitor OAT. This implies that a common strategy to reach the target INR is to monitor INR itself until it reaches the desired value, despite the amount of the administered drug. This could be a practical and economic approach but it does not consider that active warfarin and metabolites might accumulate in the blood and cause potential issues. Conversely, INR was significantly correlated with serum warfarin and 3’-hydroxywarfarin and both in turn with the weekly dosage.

With the aim of looking for alternative and efficacious strategies better conveying the true anticoagulant-burden of a patients and at a more faithful expression of the relationships between circulating drug and INR, we included in the correlation analyses, also the 3’-hydroxywarfarin active metabolite. Interestingly, 3’-hydroxywarfarin serum levels, when matched with the respective INR values, yielded a correlation degree in the global assessment (*r*^*2*^ = 0.0362; p = 0.028) with additional improvement in strength when coupled with serum warfarin (*r*^*2*^ = 0.0455; p = 0.0137). Accordingly, the strongest correlation found among the comparisons performed was that analyzing warfarin versus 3’-hydroxywarfarin concentrations (*r*^*2*^ = 0.1633; *p*<0.0001). Similarly, coupling warfarin plus 3’-hydroxywarfarin strongly improved the association with the weekly dosage (*r*^*2*^ = 0.0381; *p* = 0.024 *vs r*^*2*^ = 0.1114; *p*<0.0001, respectively). Theoretically, accounting together serum warfarin and 3’-hydroxywarfarin, better should explain INR variations or weekly dosage, than when considered separately because of both have, though at a different extent, metabolic activity. This observation could be in part explained by the fact that a given level of 3’-hydroxywarfarin entails almost established warfarin serum concentrations (being the former a metabolite of the latter). Then, 3’-hydroxywarfarin in part reflects the warfarin association with INR and adds its own degree of association with INR values. These findings, together with the strongest association found between warfarin and 3’-hydroxywarfarin, and the evidence that this latter maintains anticoagulant activity, suggest a potential value of this metabolite in the context of OAT monitoring deserving further additional investigations. In addition, the weekly drug dosage may include possible recent variations and adjustments that the metabolite alone cannot completely and instantaneously account for. Therefore, for both the above reasons this gap was offset when serum 3’-hydroxywarfarin and warfarin levels were coupled resulting in strengthened association.

A similar investigative approach was then applied to a subgroup of patients who started warfarin therapy and in which blood samples were investigated for five consecutive controls (c0-c4) till they reached the target INR. As expected, while the monitoring of OAT progressed, the mean day interval between subsequent controls increased and contextually the number of patients out-of- range decreased, reaching a value that was comparable to that of our full cohort of OAT stabilized outpatients attending the Hemostasis & Thrombosis Centre. Of note, this is a common practical approach we follow, according to the national and international guide-lines of the Federation of Centers for the Surveillance of Anticoagulant therapy (FCSA) [34].

Interestingly, by longitudinal sub-analyses aimed at revealing correlations between the effective concentration of serum warfarin and of its active metabolite 3’-hydroxywarfarin along the INR progression towards the target desirable range, we found that a mean time of 29.38±8.2 days was needed to have about 90% of patients in the target range. During this frame of time warfarin serum concentration barely correlated with INR value, showing appreciable coefficients only at the first two controls (c0 and c1), losing significant correlation in all the remaining controls (c2-c4), and showing a medium-low coefficient also in stabilized patients (*r*^*2*^ = 0.0388; *p* = 0.023). Conversely, 3’-hydroxywarfarin monitored in the same frame of time, showed better coefficients of correlation in different time-periods, better in the latest c4 control (*r*^*2*^ = 0.2157; *p* = 0.0005). It is to be taken into account that during the starting period the frequent and opposite drug adjustments strongly may affect any kind of computation. However, worth of note it was the results obtained by the multivariate analysis aimed at estimating the true 3’-hydroxywarfarin effect on INR values; it was the unique significant variable that maintained a significant role (*p* = 0.0021) in spite of warfarin that completely lost association.

For a better and more complete comprehension of the above interesting results, and on the basis of the heterogenic alleles distribution of the main genes involved in warfarin homeostasis, a genotype correlation analysis was mandatory [8,35,36].

Firstly, we stratified by the three *VKORC1* genotypes (i.e. -1173 CC, CT, TT) the findings related to warfarin and metabolite serum concentration and the amount of taken drug in the whole cohort of patients. The more interesting result was that patients with CC genotype had a significantly higher mean warfarin maintenance dosage than those with the TT genotype (*p* = 0.033). Accordingly, heterozygotes had a significant intermediate daily dose. This independent finding is in accordance with previous reports [30, 37, 38] and its validity belong to the fact that the three *VKORC1* genotype’s groups had undistinguishable mean, median and range distribution of the INR value at the day of assessment, being referred to long time established OAT patients. In addition, to determine whether the second key gene in warfarin metabolism (i.e. *CYP2C9*) may affect warfarin responsiveness by its main SNPs, we utilized the WRI and calculated the maintenance dosage. The mean of warfarin daily dose increased as the WRI category varies among the three different categories (WRI 0 vs WRI 1 + WRI 2; *p* = 0.0157) confirming the evidence that combination of *VKORC1* and *CYP2C9* gene variants strongly affect warfarin responsiveness.

## Conclusions

In conclusion, the present study demonstrates that a wide significant variation exists in warfarin dose maintenance, also in established OAT patients. By means the common practical approach, the reaching of target INR in patients starting OAT needs a long time of drug adjustment during which patients might have INR values out of range and might be exposed to potential clinical complications and risks. Approximately, more than 50% of inter-individual variation is due to SNPs in *VKORC1* and *CYP2C9* genes, and also considering age, concomitant therapy, diet and patient compliance, the remaining percentage of variations remains in part unexplained. Pharmacogenetics investigations to recognize patients *a priori* with regard to drug dosage and clinical response was recommended by FDA in 2007 for old and new drugs on based evidence, suggesting and encouraging to include also warfarin treatment [37–39]. For these reasons, only INR monitoring could be limited for dose-adjustment or for target-INR reaching. The assessment of the active serum 3’-hydroxywarfarin together with warfarin concentration may be of extraordinary help in managing patients on OAT on the basis of the finding that warfarin revealed an unexpected lost of correlation in multivariate analysis, whilst 3’-hydroxywarfarin kept very appreciable levels. Moreover, in light of the evidence that sophisticated equipments are not required, with the exception of a regular HPLC apparatus, these measurements should be included in the anticoagulant laboratories as routine practice also aimed at recognizing new active metabolites.

## Supporting Information

S1 Table Whole cohort of patients. **“**S”: undergoing therapy for long time; “C”: patients monitored since the beginning of the therapy.
(PDF)

S2 Table Patients starting anticoagulant therapy. (PDF)
