# Supplementary material for: The Active Metabolite of Warfarin (3'-Hydroxywarfarin) and Correlation with INR, Warfarin and Drug Weekly Dosage in Patients under Oral Anticoagulant Therapy: A Pharmacogenetics Study
Source: PLoS One. 2016 Sep 8;11(9):e0162084. doi: 10.1371/journal.pone.0162084 (PMC5015920; doi:10.1371/journal.pone.0162084)
Supplement: S1 Table — “S”: undergoing therapy for long time; “C”: patients monitored since the beginning of the therapy. (PDF) [file pone.0162084.s001.pdf]

S1 Table 1. Whole cohort of patients

C/S	Patient Number	INR	Warfarin (ng/mL)	3'-OH-warfarin (ng/mL)	Warfarin+3'-OH-warfarin	Warfarin weekly dosage	Cyp2C9 genotype	VKORC1 genotype
S	1	2,22	120,00	80	200,00	30	*1/*1	CC
S	2	2,19	3000,00	480	3480,00	26,25	*1/*1	CC
S	3	1,90	150,00	141	291,00	21,25	*1/*1	CC
S	4	2,64	1838,00	99	1937,00	42,5	*1/*2	CC
S	5	2,22	1068,00	598	1666,00	76,25	*1/*1	CC
S	6	1,70	810,10	112,32	922,42	41,25	*1/*2	CC
S	7	2,45	2109,00	207	2316,00	26,25	*2/*3	CC
S	8	1,81	1739,00	43	1782,00	21,25	*1/*2	CC
S	9	1,86	948,00	160	1108,00	26,25	*1/*1	CC
S	10	4,17	2601,00	1006	3607,00	52,5	*1/*1	CC
S	11	2,40	990,78	553	1543,78	60	*1/*2	CC
S	12	2,89	1810,00	863	2673,00	40	*1/*1	CC
S	13	2,41	1180,00	116	1296,00	31,25	*1/*2	CC
S	14	3,19	1524,00	276	1800,00	35	*1/*1	CC
S	15	2,15	615,00	450	1065,00	12	*1/*1	CC
S	16	2,18	2052,00	291	2343,00	45	*1/*1	CC
S	17	1,99	1285,00	128	1413,00	36,25	*1/*1	CT
S	18	2,66	1086,00	451	1537,00	17,5	*1/*2	CT
S	19	2,33	1347,00	249	1596,00	27,5	*1/*2	CT
S	20	2,73	465,00	85	550,00	8,75	*2/*3	CT
S	21	1,95	2125,00	400	2525,00	38,75	*1/*2	CT
S	22	1,92	1084,00	246	1330,00	21,25	*1/*1	CT
S	23	1,77	1805,00	396	2201,00	42,5	*1/*1	CT
S	24	3,00	2600,00	289	2889,00	15	*1/*1	CT
S	25	3,49	2266,00	827	3093,00	55	*1/*1	CT
S	26	2,18	1804,00	378	2182,00	45	*1/*1	CT
S	27	2,48	258,00	63	321,00	10	*1/*2	CT
S	28	1,58	1102,00	382	1484,00	20	*1/*1	CT
S	29	2,07	762,00	193,3587	955,36	23,75	*1/*1	CT
S	30	2,46	1172,26	186	1358,26	30	*1/*1	CT
S	31	2,15	1138,00	73	1211,00	28,75	*1/*1	CT
S	32	1,92	773,00	30	803,00	16,25	*1/*1	CT
S	33	2,33	1128,00	283	1411,00	21,25	*1/*1	CT
S	34	2,63	1015,00	84	1099,00	35	*1/*1	CT
S	35	1,79	768,00	149	917,00	26,25	*1/*1	CT
S	36	1,57	1182,00	127	1309,00	41,25	*1/*1	CT
S	37	1,88	30,00	164,72	194,72	37	*1/*1	CT
S	38	2,02	1080,00	101	1181,00	27,5	*1/*1	CT
S	39	2,33	1892,00	191	2083,00	25	*1/*1	CT
S	40	1,61	751,00	210	961,00	12,5	*1/*2	TT
S	41	3,24	1243,00	137	1380,00	20	*1/*2	TT
S	42	2,71	660,00	238	898,00	12,5	*1/*1	TT
S	43	2,00	953,06	221	1174,06	12,5	*1/*2	TT
S	44	2,19	3440,00	490	3930,00	40	*1/*1	TT
S	45	2,41	230,00	200	430,00	8,75	*1/*1	TT
S	46	1,77	927,00	157	1084,00	18,75	*1/*1	TT
S	47	2,29	2300,00	320	2620,00	15	*1/*2	TT

S	48	2,17	960,00	78	1038,00	12,5	*1/*3	TT
S	49	2,07	684,00	56	740,00	20	*1/*1	TT
S	50	2,44	1162,73	427	1589,73	20	*1/*1	TT
S	51	2,90	3370,00	100	3470,00	26,25	*1/*1	TT
S	52	2,80	677,28	62,14	739,42	11,25	*1/*1	CT
S	53	3,43	1480,52	74,4	1554,92	26,25	*1/*1	TT
S	54	2,37	372,63	655,08	1027,71	60	*1/*1	TT
S	55	2,11	1280,07	199	1479,07	58,75	*1/*1	TT
S	56	2,23	1070,43	55,78	1126,21	28,75	*1/*1	TT
S	57	1,99	1297,23	107,74	1404,97	15	*1/*2	TT
S	58	2,85	945,49	64,29	1009,78	17,5	*1/*1	CC
S	59	2,85	643,12	190,25	833,37	26,25	*1/*1	CC
S	60	3,80	1061,72	174,18	1235,90	30	*1/*2	CT
S	61	2,37	1190,58	84,71	1275,29	33,75	*1/*1	CC
S	62	2,43	810,50	114,86	925,36	28,75	*1/*2	CT
S	63	2,37	985,75	37,06	1022,81	5	*1/*1	CT
S	64	2,03	903,14	156	1059,14	51,25	*1/*1	CT
S	65	3,78	1408,83	339	1747,83	32,5	*1/*1	CT
S	66	2,71	1143,40	177,5	1320,90	32,5	*1/*1	TT
S	67	3,00	741,52	35,37	776,89	23,75	*2/*3	TT
S	68	1,99	1095,53	34,89	1130,42	31,25	*1/*2	CT
S	69	3,73	1422,92	300	1722,92	25	*1/*1	CT
S	70	1,91	746,21	30,4	776,61	32,5	*1/*3	CT
S	71	2,99	1135,97	27,76	1163,73	22,5	*1/*2	CT
S	72	2,96	32,12	33,44	65,56	21	*1/*1	CT
S	73	3,00	1280,18	33,82	1314,00	16,25	*1/*1	CT
S	74	2,29	726,84	239,12	965,96	22,5	*1/*1	CC
S	75	2,37	435,66	217,32	652,98	7,5	*1/*1	CC
S	76	2,47	1452,96	183,76	1636,72	67,5	*1/*1	CC
S	77	3,61	1500,84	505,58	2006,42	31,25	*1/*1	CC
S	78	3,00	602,52	26,29	628,81	16,25	*1/*1	CT
S	79	2,20	115,00	77	192,00	28	*1/*1	CC
S	80	1,85	146,00	139	285,00	20	*1/*1	CC
S	81	2,5	440	450	890,00	70	*1/*1	CC
C	82	2,09	378,27	101,38	479,65	36,9	*1/*1	CC
C	83	2,70	1750,00	517	2267,00	36,16	*1/*1	CC
C	84	2,45	582,51	50,04	632,55	25,4	*1/*1	CC
C	85	2,80	1177,00	170	1347,00	27,58	*1/*1	CC
C	86	2,00	1427,00	167	1594,00	31,67	*1/*1	CC
C	87	2,80	1752,00	490	2242,00	8,75	*1/*1	CC
C	88	2,58	1684,00	241	1925,00	25,48	*1/*1	CC
C	89	2,40	750,00	230	980,00	26,25	*1/*1	CC
C	90	2,13	956,00	11,52	967,52	31,57	*1/*1	CC
C	91	1,99	316,77	7	323,77	30,17	*1/*1	CC
C	92	2,40	900,00	197,1	1097,10	28,33	*1/*3	CC
C	93	2,20	738,62	321,5	1060,12	59,57	*1/*1	CC
C	94	2,29	1248,00	254	1502,00	32,42	*1/*2	CT
C	95	1,99	948,29	185,89	1134,18	25,2	*1/*2	CT
C	96	2,90	900,00	285	1185,00	17,5	*1/*1	CT
C	97	3,00	1500,00	395	1895,00	7,1	*1/*1	CT
C	98	2,60	1000,00	160	1160,00	25,45	*1/*1	CT
C	99	2,90	240,00	285	525,00	23,92	*1/*1	CT
C	100	2,70	750,00	185	935,00	21	*1/*1	CT

C	101	2,70	310,00	150	460,00	17,5	*1/*1	CT
C	102	2,70	800,00	85	885,00	13,75	*1/*1	CT
C	103	2,25	1157,20	211,11	1368,31	27,5	*1/*1	CT
C	104	3,00	1649,00	286,77	1935,77	31,72	*1/*1	CT
C	105	2,40	860,00	220	1080,00	24,87	*1/*2	CT
C	106	1,99	500,00	100	600,00	27,5	*1/*1	CT
C	107	2,25	700,00	85	785,00	26,25	*1/*1	CT
C	108	2,30	800,00	192,42	992,42	24,89	*1/*1	CT
C	109	2,69	492,00	74,83	566,83	30,99	*1/*1	CT
C	110	2,20	410,08	116	526,08	32,66	*1/*3	TT
C	111	3,16	450,00	25	475,00	24,06	*1/*1	TT
C	112	2,58	1229,00	326	1555,00	21,11	*1/*1	TT
C	113	2,56	689,89	178,119	868,01	26,67	*1/*2	TT
C	114	2,30	943,53	184,95	1128,48	21,33	*1/*1	TT
C	115	2,47	400,00	90	490,00	13,13	*1/*1	TT
C	116	2,60	1220,00	227,92	1447,92	10	*2/*3	TT
C	117	2,15	400,00	250	650,00	17,5	*1/*1	TT
C	118	1,80	713,11	240,59	953,70	20	*1/*2	CT
C	119	2,08	594,57	15	609,57	10	*1/*1	CC
C	120	3,24	1332,48	357,4	1689,88	35	*1/*2	CC
C	121	2,50	545,00	52	597,00	26,25	*1/*2	CC
C	122	1,54	346,98	205,13	552,11	13,75	*1/*1	CC
C	123	1,68	717,01	88,58	805,59	23,75	*1/*1	CT
C	124	2,43	671,98	113,19	785,17	26,25	*1/*1	CC
C	125	1,50	375,80	39,68	415,48	15	*1/*1	CT
C	126	1,50	280,85	30,48	311,33	17,5	*1/*1	CC
C	127	2,31	442,06	11,46	453,52	20	*1/*2	CT
C	128	2,19	751,05	106,21	857,26	11,25	*1/*1	CT
C	129	3,22	871,83	105,43	977,26	20	*1/*1	CC
C	130	1,97	1056,94	38,27	1095,21	18,75	*1/*2	CT
C	131	3,13	3545,67	383,47	3929,14	15,41	*1/*1	TT
C	132	3,34	30	310,43	340,43	9	*1/*1	CC
C	133	3,34	35	280	315,00	9	*1/*1	CC
