# Supplementary material for: The Active Metabolite of Warfarin (3'-Hydroxywarfarin) and Correlation with INR, Warfarin and Drug Weekly Dosage in Patients under Oral Anticoagulant Therapy: A Pharmacogenetics Study
Source: PLoS One. 2016 Sep 8;11(9):e0162084. doi: 10.1371/journal.pone.0162084 (PMC5015920; doi:10.1371/journal.pone.0162084)
Supplement: S2 Table — (PDF) [file pone.0162084.s002.pdf]

S2 Table 1. Patients starting anticoagulant therapy

Patient Number	INR c0	INR c1	INR c2	INR c3	INR c4	Warfarin (ng/mL) c0	Warfarin (ng/mL) c1	Warfarin (ng/mL) c2	Warfarin (ng/mL) c3	Warfarin (ng/mL) c4	3-OH-warfarin (ng/mL) c0	3-OH-warfarin (ng/mL) c1	3-OH-warfarin (ng/mL) c2	3-OH-warfarin (ng/mL) c3	3-OH-warfarin (ng/mL) c4
82	1,23	1,41	1,91	2,13	2,09	1073,86	443,11	457,74	469,33	378,27	362,61	113,69	104,22	93,03	101,38
83	1,22	1,2	1,32	2,33	2,7	457	854	854,7	1980	1750	24	112,09	145,8	527	517
84	1,07	1,38	2,33	1,9	2,45	0	495	500	518,94	582,51	0	51	53	55,73	50,04
85	0,98	1,07	1,66	2,47	2,8	84	509,89	791,04	1177	1177	64	127	155,06	168	170
86	1,12	1,21	1,2	1,75	2	327	576,6	648	924	1427	63	84	112,09	163,47	167
87	1,46	1,35	2,18	2,61	2,8	428	643,32	1245,32	1752	1752	54	42	203,63	518	490
88	1,01	1,06	1,19	1,49	2,58	324	527,4	567,07	710,03	1684	24,2	99,01	178	321	241
89	1,5	2,4	2,47	2,5	2,4	870,83	850	805,9	805,9	750	226,8	224	242,8	242,8	230
90	1	1,18	1,5	2,64	2,13	0	352,53	479,34	620,34	956	0	33,1	21,55	17,81	11,52
91	1,06	1,26	1,7	2,34	1,99	0	175	195,68	296,12	316,77	0	6	7,31	6,22	7,7
92	1,32	2,04	2,11	2,25	2,4	591,3	856,4	1026	1026	900	98,4	190,56	197,1	197,1	197,1
93	1,09	1,67	1,92	2,14	2,2	328,46	854,3	914,94	810,1	738,62	43,58	155,99	389	254,3	321,5
94	1,09	1,46	1,85	2,22	2,29	481	257	695,4	1057,9	1248	24	13	154	221	254
95	1,13	1,71	1,61	2,17	1,99	519	1853	654	1039	948,29	85	159,73	150,39	147	185,89
96	1,21	1,49	1,85	2,13	2,9	250	900	1003	1003	900	0	300	293	293	285
97	1,4	1,86	2,35	2,45	3	746,6	886,35	1796	1796	1500	130,77	154,34	452	452	395
98	1,13	1,21	1,51	2,12	2,6	734	576,6	719,56	1154	1000	128,4	158,03	141,05	157	160
99	1,18	1,47	2,03	2,68	2,9	25	204	422	271	240	15	124	139	289	285
100	1,31	1,39	1,86	2	2,7	126	662,38	886,35	886,35	750	75	129,84	173,74	173,74	185
101	1,03	1,25	1,34	2,96	2,7	200	313	425	500	310	107	149	215	250	150
102	1,5	1,79	2,25	2,5	2,7	999	826,3	1033,3	1033,3	800	67,4	62,6	84,6	84,6	85
103	1,2	1,77	2,55	1,85	2,25	249,7	357,4	435,8	328,4	1157,2	42,8	127,5	238,2	172,81	211,11
104	1,45	1,35	1,5	1,66	3	854,3	643,32	714,8	791,04	1649	149,3	265,3	498,6	328,4	286,77
105	1,09	3,06	2,03	2,25	2,4	1258	1124,82	967	900	860	84,5	279,3	224	245	220
106	1,17	3,64	1,56	1,59	1,99	781	550	458	458	500	77	85	77	77	100
107	1,03	1,08	1,4	1,9	2,25	490,83	624,33	756	756	700	96,21	100,88	85	85	85
108	1,45	1,26	2,06	2,25	2,3	496,2	724,58	994	994	800	125	117,7	192,42	192,42	192,42
109	1,08	1,67	2,28	3,55	2,69	0	126,39	254,6	364,15	492	0	103	83,71	14,62	74,83
110	1,01	1,03	1,68	2,12	2,2	0	240	250,748	302,84	410,08	0	90	108,87	128,87	116

111	0,99	1,29	2,93	2,99	3,16	0	265,57	270,08	482,14	450	0	15,23	6,8	31,63	25
112	0,99	1,11	1,91	1,77	2,58	324,5	725,4	910,17	1245,7	1229	68	103,69	215,4	165,34	326
113	1,08	1,5	2,58	3,53	2,56	319,82	137,25	423,49	735,95	689,89	199,9633	2294,64	2130,297	2327,4	178,119
114	0,98	1,34	1,98	2,21	2,3	493,2	458,32	862	934	943,53	65,4	125,17	215	196,48	184,95
115	1,08	1,7	1,76	1,8	2,47	38	425	410	410	400	73	93	100	100	90
116	1,03	3,43	1,87	2,44	2,6	391,5	734,6	753,4	1379	1220	68	320,4	174,68	227,92	227,92
117	1,18	1,88	1,2	1,9	2,15	549,32	794,66	571,84	571,84	400	75,6	175,61	249	249	250
118	1,13	1,62	2,72	1,86	1,8	0	524,32	427,36	631,68	713,11	0	63,24	43,32	60,92	240,59
119	1,19	1,32	4,97	1,47	2,08	0	453,94	697,94	330,76	594,57	0	7,73	3,06	14,68	15
120	1,17	1,22	1,61	2,13	3,24	0	460,26	878,26	1081,5	1332,48	0	67,8	78,72	53,28	357,4
121	1,11	1,53	2,06	2,4	2,5	0	401,12	591,3	550	545	0	52,28	40,04	54	52
122	1,11	1,8	1,95	1,83	1,54	0	823,42	756,28	740,38	346,98	0	64,32	46,57	53,29	205,13
123	1,15	1,2	1,32	1,47	1,68	0	204,35	357,35	511,63	717,01	0	55,73	67,36	86,1	88,58
124	1,24	1,27	1,61	2,59	2,43	0	268,81	545,77	981,98	671,98	0	116,19	110,39	141,35	113,19
125	1,18	2,57	3	2,51	1,5	0	584,57	493,61	636,73	375,8	0	173,44	106,27	73,51	39,68
126	1,13	3,16	2,36	2,2	1,5	0	586,47	446,08	353,19	280,85	0	13,35	21,91	60,81	30,48
127	0,99	1,56	1,46	2,22	2,31	0	364,68	216,75	411,47	442,06	0	9,08	2,83	9,41	11,46
128	1,13	3,17	2,33	2,46	2,19	0	1061,88	2847,01	2649,92	751,05	0	232,2	2192,16	1106,93	106,21
129	1,01	1,48	3,11	2,34	3,22	0	397,09	721,01	590,16	871,83	0	104,67	127,43	92,13	105,43
130	1,5	2,29	1,77	2,35	1,97	125	980,72	715,17	923,52	1056,94	22	37,74	28,67	47,95	38,27
131	1,14	3,34	5,73	4,01	3,13	0	3356,1	4187,17	3669,52	3545,67	0	663,81	748,43	406,21	383,47
132	1,14	1,9	1,95	2,5	3,34	0	123	180	120	30	0	38,2	45	51,06	310,43
133	1,18	1,7	2	2,4	3,34	0	110	190	180	35	0	33,03	40,12	58,02	280
